# Fatty acid transport protein 2 inhibition enhances glucose tolerance through **α** cell–mediated GLP-1 secretion

**DOI:** 10.1172/JCI192011

**Published:** 2025-09-16

**Authors:** Shenaz Khan, Robert J. Gaivin, Zhiyu Liu, Vincent Li, Ivy Samuels, Jinsook Son, Patrick Osei-Owusu, Jeffrey L. Garvin, Domenico Accili, Jeffrey R. Schelling

**Affiliations:** 1 Department of Physiology and Biophysics, Case Western Reserve University School of Medicine, Cleveland, Ohio, USA.; 2 Louis Stokes Cleveland VA Medical Center, VA Northeast Ohio Healthcare System; Department of Ophthalmic Research, Cole Eye Institute, Cleveland, Ohio, USA.; 3 Department of Medicine and Naomi Berrie Diabetes Center, Vagelos College of Physicians and Surgeons of Columbia University, New York, New York, USA.; 4 Department of Medicine, Case Western Reserve University School of Medicine, Cleveland, Ohio, USA.

**Keywords:** Endocrinology, Metabolism, Beta cells, Glucose metabolism, Insulin

## Abstract

Type 2 diabetes affects more than 38 million people in the United States, and a major complication is kidney disease. During the analysis of lipotoxicity in diabetic kidney disease, global fatty acid transport protein 2 (FATP2) gene deletion was noted to markedly reduce plasma glucose in db/db mice due to sustained insulin secretion. To identify the mechanism, we observed that islet FATP2 expression was restricted to α cells and that α cell FATP2 was functional. Basal glucagon and alanine-stimulated gluconeogenesis were reduced in FATP2-KO db/db mice compared with db/db mice. Direct evidence of FATP2-KO–induced α cell–mediated glucagon-like peptide 1 (GLP-1) secretion included increased GLP-1^+^ α cell mass in FATP2-KO db/db mice, small-molecule FATP2 inhibitor enhancement of GLP-1 secretion in αTC1-6 cells and human islets, and exendin[9-39]-inhibitable insulin secretion in FATP2 inhibitor–treated human islets. FATP2-dependent enteroendocrine GLP-1 secretion was excluded by demonstration of similar glucose tolerance and plasma GLP-1 concentrations in db/db FATP2-KO mice following oral versus i.p. glucose loading, nonoverlapping FATP2 and preproglucagon mRNA expression, and lack of FATP2 and GLP-1 coimmunolocalization in the intestines. We conclude that FATP2 deletion or inhibition exerts glucose-lowering effects through α cell–mediated GLP-1 secretion and paracrine β cell insulin release.

## Abstract 2

Deletion or inhibition of FATP2 markedly reduced plasma glucose in diabetic mice by enhancing α-cell GLP-1 secretion, and paracrine stimulation of ß-cell insulin release.

## Graphical abstract

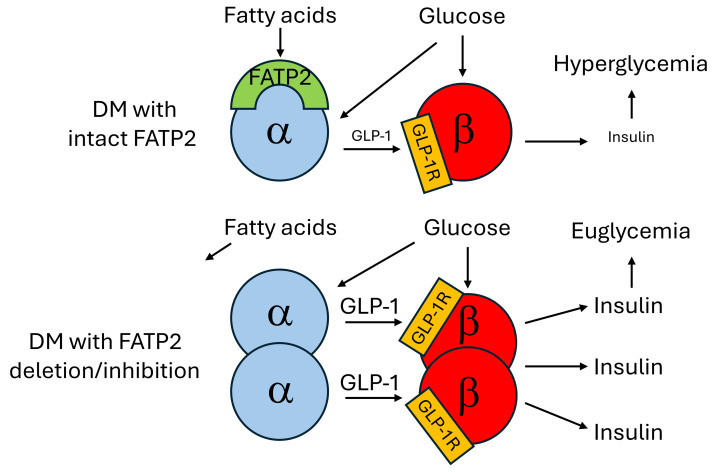

## Introduction

Type 2 diabetes affects more than 38 million people in the United States (830 million people worldwide) and is a major public health problem due to morbidity and mortality from complications at the microvascular (kidney disease, retinopathy, neuropathy) and macrovascular (myocardial infarction, stroke, peripheral vascular) levels. The pathophysiologic mechanisms are complex, with substantial contributions from altered glucose and lipid metabolism (1, 2).

The lipid abnormalities in diabetes include increased plasma fatty acid concentrations (3). Fatty acids circulate predominantly as noncovalently bound complexes with albumin or as covalently linked esters with glycerol to form triglycerides. Cellular fatty acid uptake is facilitated by a family of 6 evolutionarily conserved plasma membrane fatty acid transport proteins (FATP1–6), which are expressed in a tissue-specific fashion (4).

FATP2 is a major fatty acid transporter in liver and kidney and has been implicated in the pathophysiology of metabolic dysfunction–associated steatotic liver disease (MASLD) and diabetic kidney disease (5). A modest reduction of fasting plasma glucose levels was observed in mice with FATP2 gene (*Slc27a2*) deletion (6). Inhibition of FATP2 by siRNA tail vein injection in high-fat diet–induced diabetic mice also resulted in mild plasma glucose reductions, as well as improved insulin sensitivity (7). Because tail vein–injected siRNA uptake is primarily by liver (8), it was assumed that the hypoglycemic effect of FATP2 inhibition was mediated by enhanced hepatic glucose metabolism. However, tail vein–injected reporter siRNAs are detectable in other FATP2-expressing organs, including intestine and pancreas (9, 10), which raises the possibility that extrahepatic FATP2 inhibition contributes to the glucose-lowering phenotype.

In contrast to the modest glucose reduction with liver FATP2 inhibition (7), global FATP2 deletion is associated with profoundly lower plasma glucose in genetic and inducible mouse models of type 2 diabetes (11). Furthermore, diabetic mice with intact FATP2 develop reduced plasma insulin levels, whereas diabetic mice with FATP2 deletion demonstrate islet hypertrophy and sustained hyperinsulinemia (11). These observations suggest that FATP2 inhibition enhances pancreatic β cell mass and function. Localization of FATP2 to specific pancreatic cells and assignment of FATP2 inhibition to pancreatic endocrine functions, have not, to our knowledge, been previously described.

Glucose-stimulated insulin secretion (GSIS) is augmented by glucagon-like peptide 1 (GLP-1) in diabetes (12). Following a glucose- or fat-containing meal, GLP-1 is secreted into the circulation by enteroendocrine L cells in the distal ileum and proximal colon and ultimately binds to GLP-1 receptors on pancreatic β cells to stimulate insulin secretion (13). Fatty acids can also directly stimulate GSIS through binding to free fatty acid receptor 1 (FFAR1), which is expressed at low levels on β cells (14, 15). The insulinotropic effects of fatty acids in acute models are counterbalanced by chronic fatty acid–induced α and/or β cell desensitization and decreased insulin secretion (16), which may be mediated by specific fatty acid receptors.

Pancreatic α cells also secrete GLP-1, which enhances GSIS through paracrine activation of the β cell GLP-1 receptor (12, 15, 17), particularly under conditions of β cell stress (18). The primacy of paracrine GLP-1 is supported by observations that GLP-1 secreted by enteroendocrine cells has a half-life of only 2 minutes (19), due to proteolysis by local dipeptidyl peptidase 4 (DPP-4). It has therefore been postulated that gut-derived GLP-1 may not achieve sufficient concentration to stimulate distant β cell GLP-1 receptors, and that an α cell, rather than an enteroendocrine source of GLP-1, regulates insulin secretion under diabetic conditions (18).

With the emergence of GLP-1 receptor agonists as weight loss drugs, there is recent intense interest regarding GLP-1 regulation of lipid metabolism. However, the effect of fatty acids on GLP-1 biology is much less well understood, and the influence of FATP2 inhibition on GLP-1 pathways has not been investigated. In this report, we describe the mechanisms of insulinotropic activity by FATP2 inhibition through augmentation of α cell–mediated GLP-1 secretion.

## Results

Diabetic mice with global FATP2 gene deletion (FATP2-KO) developed markedly reduced fasting plasma glucose (11). FATP2 is most abundantly expressed in the kidney and, within the kidney, exclusively in the apical proximal tubule membrane (20, 21). The proximal tubule contributes to gluconeogenesis, particularly in the pathogenesis of diabetes (22). However, deletion of proximal tubule FATP2 (Supplemental Figure 1; supplemental material available online with this article; https://doi.org/10.1172/JCI192011DS1) in an inducible model of diabetes did not alter fasting plasma glucose concentrations (Supplemental Figure 2). These data suggest that the glucose-lowering effect in global FATP2-KO mice was not due to proximal tubule FATP2 gene deletion but rather to an extra-renal mechanism.

**Figure 1:**
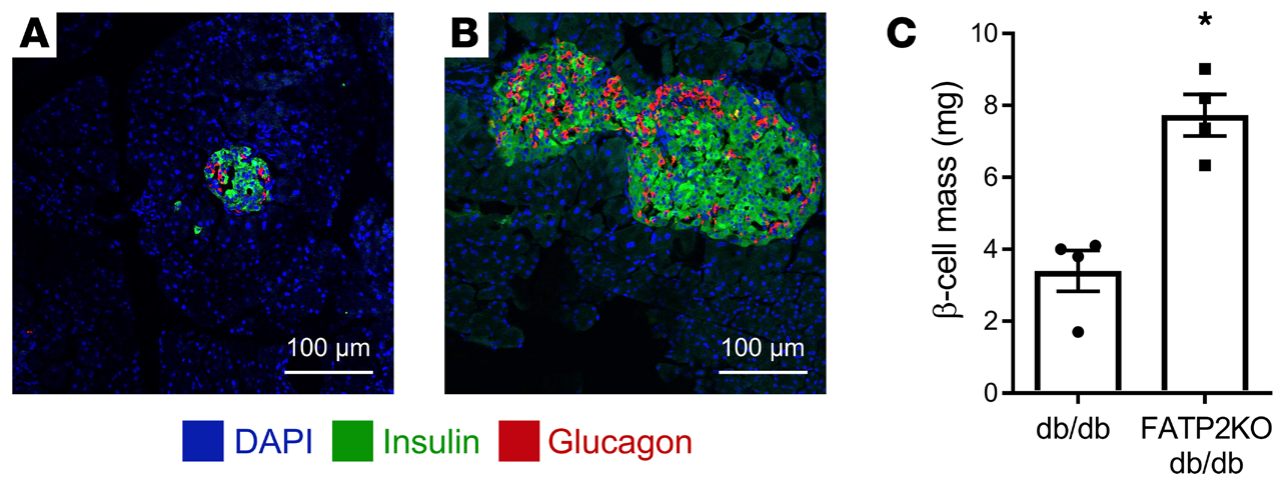
Islet hypertrophy and increased β cell mass in FATP2-KO db/db mice. Representative IHC images of pancreatic islets from db/db (**A**) and FATP2-KO db/db (**B**) mice. As described in Methods, α and β cells were labeled with glucagon and insulin antibodies, respectively. Scale bars: 100 μm. (**C**) β Cell mass was calculated as described in Methods for db/db and FATP2-KO db/db mice. Data represent the mean ± SEM. *P < 0.01, by Student’s *t* test.

Pancreatic islet FATP2 protein expression is upregulated in the setting of elevated glucose concentration (23), and global FATP2 gene deletion in diabetic mice is associated with increased islet area and sustained plasma insulin levels (11), suggesting that inhibition of FATP2 mediates the protection of pancreatic islet function. Compared with diabetic *Lepr^db/db^* (db/db) mice with intact FATP2, we observed that FATP2-KO db/db mice had islet hypertrophy (Figure 1, A and B, respectively) and increased β cell mass (Figure 1C). These data are consistent with FATP2 deletion causing the rescue of β cell failure in db/db mice (11, 24).

**Figure 2:**
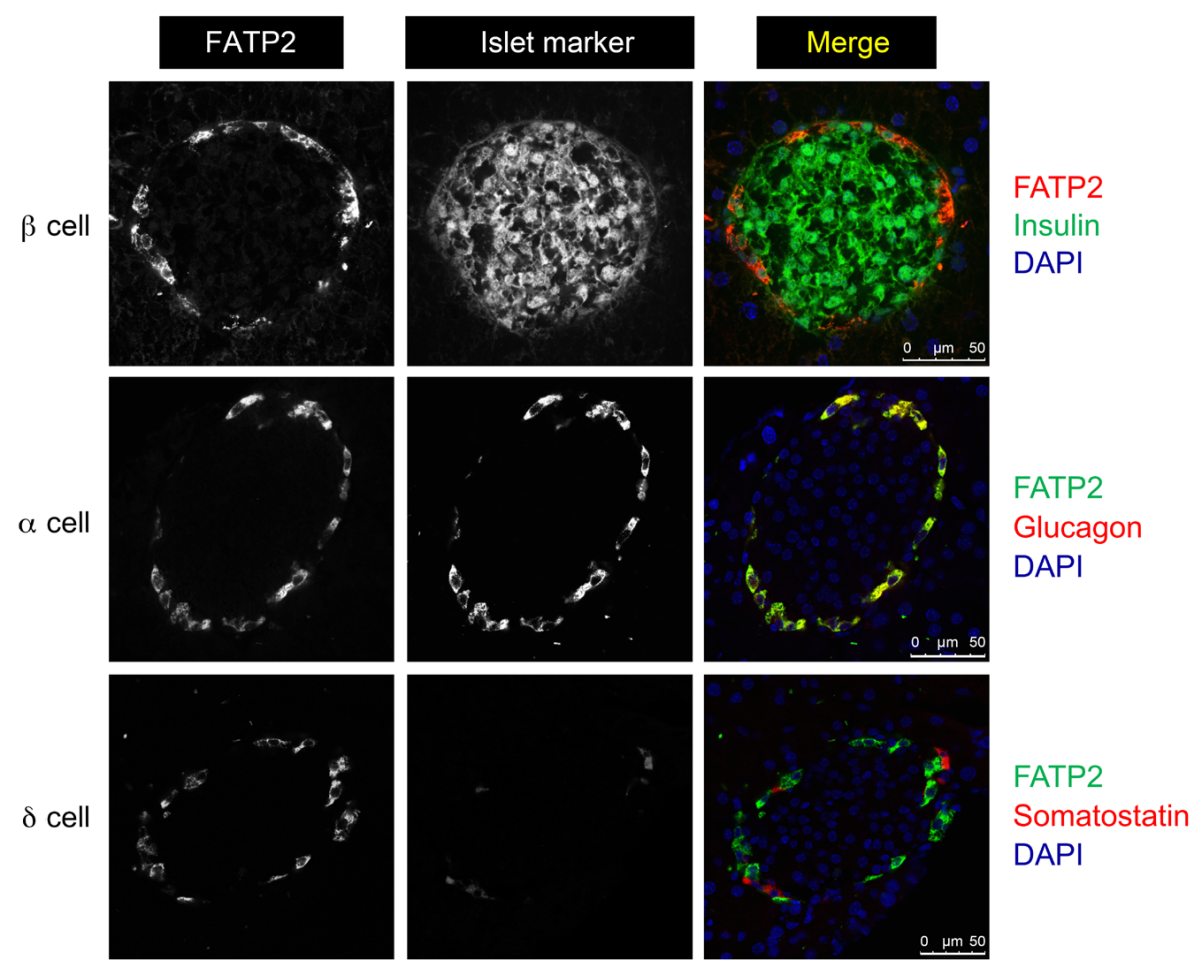
FATP2 expression localizes to pancreatic α cells in mouse islets. WT mouse pancreatic islets were immunohistochemically labeled for FATP2 expression in α, β, and δ cells as described in Methods. Merged images, representing cell-specific FATP2 expression, are shown in yellow. Micrometer scale bars are shown at the bottom right of each merged image.

**Figure 3:**
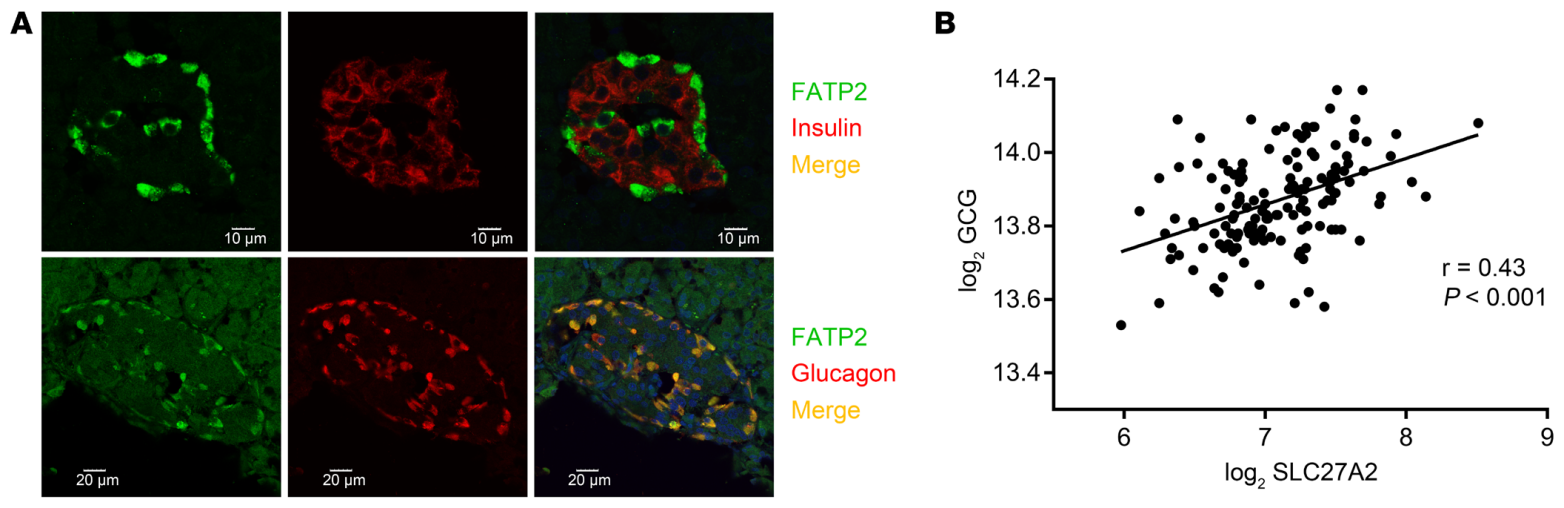
FATP2 expression localizes to pancreatic α cells in human islets. (**A**) Paraffin sections of human pancreas were immunohistochemically labeled for FATP2 expression in α and β cells as described in Methods. Merged images, representing cell-specific FATP2 expression, are shown in yellow. Micrometer scale bars are shown at the bottom of each image. (**B**) Gene expression correlation between *GCG* and FATP2 gene (*SLC27A2*) from 2 public normal human islet transcriptome datasets (GSE38642 and GSE50397) using online software (http://r2.amc.nl). Data were analyzed by linear regression and Pearson correlation.

**Figure 4:**
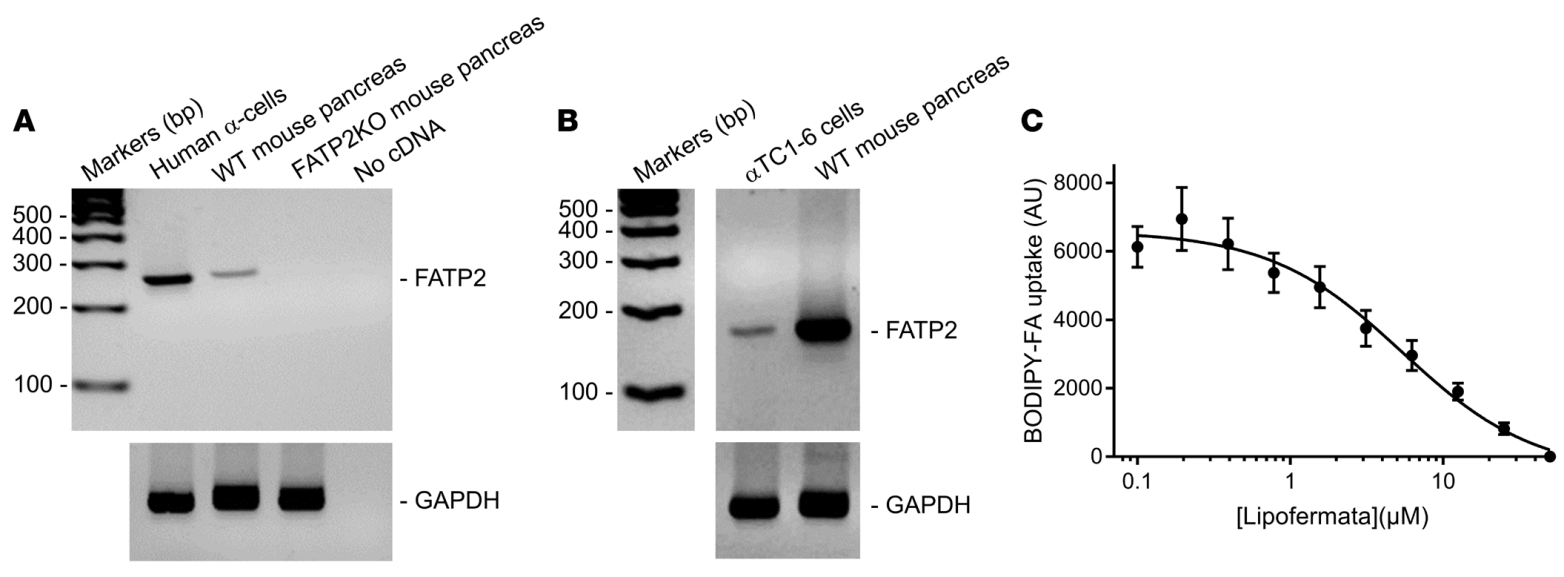
FATP2 expression and function in α cells. (**A** and **B**) FATP2 and loading control GAPDH mRNA expression was determined in human and mouse pancreatic tissue and α cell lines by RT-PCR (as described in Methods). Data are representative of 3 experiments per condition. (**C**) Mouse αTC1-6 cells were preincubated with lipofermata (1 hour, 37°C, 0–50 μM) in triplicate. BODIPY-labeled fatty acid uptake velocity was then determined, as described in Methods. Results show the mean ± SEM of 4 experiments.

*Slc27a2* mRNA is expressed in the pancreas (11) and variably in α and β cells from single-cell RNA-Seq databases (25–30). Figure 2 demonstrates that FATP2 protein colocalized exclusively with α, but not β or δ, cells in mice. Figure 3A demonstrates similar colocalization of FATP2 with α cells in human pancreas, a finding that is consistent with a correlation between *SLC27A2* and *GCG* (preproglucagon gene), which contains protein coding sequence for glucagon and GLP-1 (Figure 3B). *SLC27A2 and Slc27a2* mRNA is expressed in human and mouse and human pancreatic tissue and α cells (Figure 4, A and B) but is undetectable in rat insulinoma INS-1 β cells (not shown). Mouse pancreas and αTC1-6 cells predominantly express the Fatp2a variant (Supplemental Figure 3), which contains acyl CoA synthetase activity within the cytosolic domain (31) and in a plasma membrane distribution (Supplemental Figure 4). *Slc27a2* mRNA expression was increased in islets from db/db mice compared with WT mice, although the difference was not significant (Supplemental Figure 5). To determine whether α cell FATP2 is functional, we measured long-chain fatty acid transport in αTC1-6 cells. We found that fatty acid uptake was blocked by the FATP2 inhibitor lipofermata (Figure 4C). The IC_50_ value (5.4 μM) is in agreement with that for other epithelial cells (32, 33). We conclude from these experiments that α cells express functional FATP2, which is sustained with diabetes.

**Figure 5:**
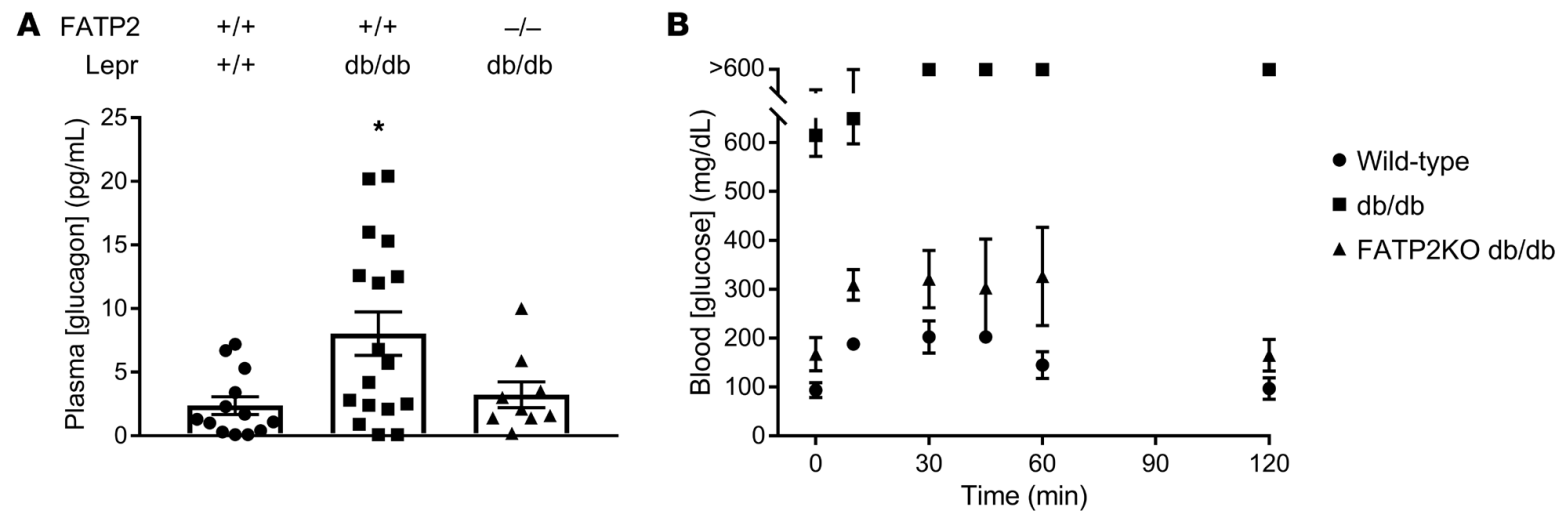
Effects of FATP2 deletion on glucagon. (**A**) Random (nonfasting) plasma glucagon concentrations in 4- to 6-month-old WT and db/db mice with or without FATP2 gene deletion. Each symbol in the scatter bars represents the mean from 1 sample assayed in duplicate (*n* = glucagon concentrations from 9–17 mice per genotype). *P < 0.05 compared with WT by ANOVA with Tukey’s post hoc test for multiple comparisons. (**B**) Serial glucose measurements in 4- to 6-month-old WT and db/db mice with or without *FATP2* gene deletion (*n* = 3 mice per group) following alanine administration (2 g/kg i.p.). Data represent the mean ± SEM.

We next focused on the mechanism by which α cell FATP2 deletion regulates insulin secretion. GLP-1 and glucagon bind with high and low affinity, respectively, to the GLP-1 receptor on β cells, which facilitates GSIS (12). Random (nonfasting) plasma glucagon levels were increased in db/db mice with intact FATP2, decreased in FATP2-KO db/db mice, and not significantly different compared with WT glucagon levels (Figure 5A), suggesting that glucagon was not the stimulus for sustained insulin secretion in FATP2-KO db/db mice. To address the effects of FATP2 deletion on glucagon-induced hepatic gluconeogenesis, we conducted alanine tolerance tests in fasted db/db versus FATP2-KO db/db mice. Figure 5B demonstrates transient alanine-stimulated glucose increases in WT and FATP2-KO db/db mice, whereas db/db mice experienced sustained hyperglycemia (>600 mg/dL) from 30–120 minutes. The data suggest that the relatively modest effect on glucose in FATP2-KO db/db mice reflects reduced glucagon-stimulated gluconeogenesis.

**Figure 6:**
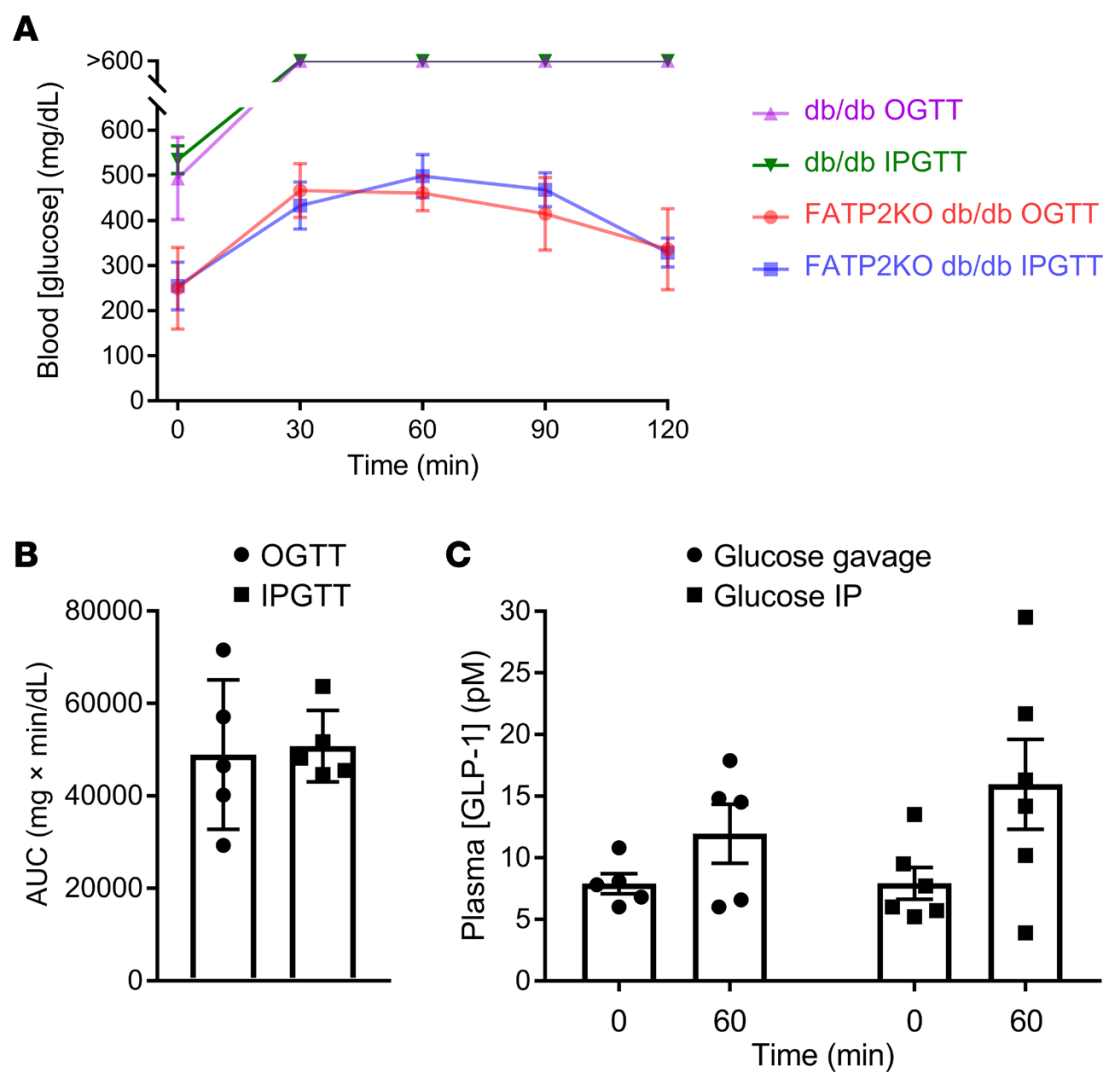
Blood glucose and plasma GLP-1 concentrations following OGTTs and IPGTTs. (**A**) OGTTs and IPGTTs were conducted in db/db and FATP2-KO db/db mice, as described in Methods. Blood glucose levels were determined at the indicated times in 5 mice per group. (**B**) As an index of glucose disposal, the AUC corresponding to FATP2-KO db/db experiments in **A** was integrated using GraphPad Prism 7 software. (**C**) Plasma was obtained at baseline and at the 1-hour time point during the OGTTs or IPGTTs in FATP2-KO db/db mice. Data represent the mean ± SEM.

We next focused on the effect of FATP2 gene deletion on GLP-1. The relative contribution of enteroendocrine L cell– versus α cell–derived GLP-1 on β cell insulin secretion has been debated (34). To investigate whether enteroendocrine cells are the GLP-1 source in FATP2-KO db/db mice, oral glucose tolerance tests (OGTTs) and intraperitoneal glucose tolerance tests (IPGTTs) were conducted in db/db mice with or without FATP2 gene deletion. The rationale is if the major GLP-1 source is enteroendocrine, oral glucose would stimulate a greater increase in plasma GLP-1 and superior glucose tolerance compared with i.p. glucose (35). Four-month-old db/db and FATP2-KO db/db mice were obese, although baseline weights (45 ± 9 g and 54 ± 10 g, respectively) were similar (*P* > 0.05). Figure 6A shows markedly lower fasting plasma glucose concentrations in FATP2-KO db/db compared with db/db mice, consistent with previous reports (11). Plasma glucose values were higher than 600 mg/dL in all db/db mice during the OGTT and IPGTT at 30–120 minutes. Figure 6, A and B, shows no difference between OGTTs and IPGTTs for FATP2-KO db/db mice. Glucose disposal was also similar following OGTTs versus IPGTTs in nondiabetic FATP2-KO mice (Supplemental Figure 6). Importantly, plasma GLP-1 increases were similar in FATP2-KO db/db mice after oral and i.p. glucose loading (Figure 6C). The lack of enhanced glucose tolerance and GLP-1 concentration with oral glucose suggests that α cells, rather than enteroendocrine L cells, were the source of GLP-1 in mice with global FATP2 gene deletion.

**Figure 7:**
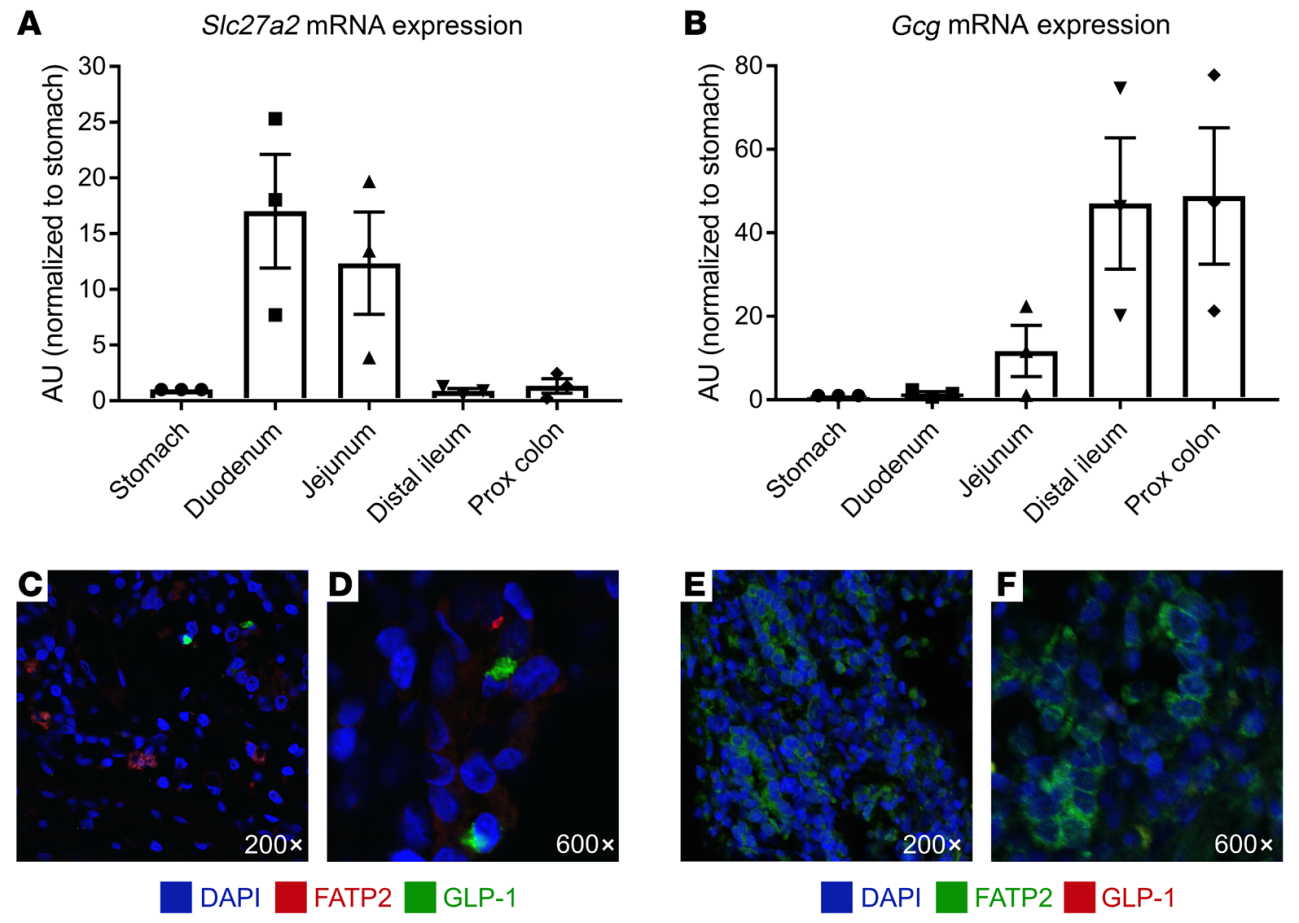
FATP2 and GLP-1 localization in the intestines. *Slc27a2* (**A**) and *Gcg* (**B**) mRNA expression levels were determined in mouse gut segments by qPCR, as described in Methods. Data were normalized to expression in stomach, which was defined as 1.0. Immunohistochemical labeling of FATP2 and GLP-1 in human distal ileum (**C** and **D**) (note that FATP2 is red and GLP-1 is green) and duodenum (**E** and **F**) (note that FATP2 is green and GLP-1 is red). Representative images from 5 mice are shown. Original magnification, ×200 (**C** and **E**) and ×600 (**D** and **F**). Data represent the mean ± SEM.

To further address the possible FATP2 effects on enteroendocrine GLP-1 secretion, we evaluated *Slc27a2* and *Gcg* mRNA expression in intestine segments by quantitative PCR (qPCR). Both transcripts were detected throughout the mouse gastrointestinal (GI) tract, but with distinct patterns and minimal overlap (Figure 7, A and B). Protein expression in human distal ileum (Figure 7, C and D) and duodenum (Figure 7, E and F) demonstrated no colocalization of FATP2 and GLP-1. Taken together, the data suggest that FATP2 deletion did not directly influence GLP-1 synthesis or secretion by enteroendocrine cells.

**Figure 8:**
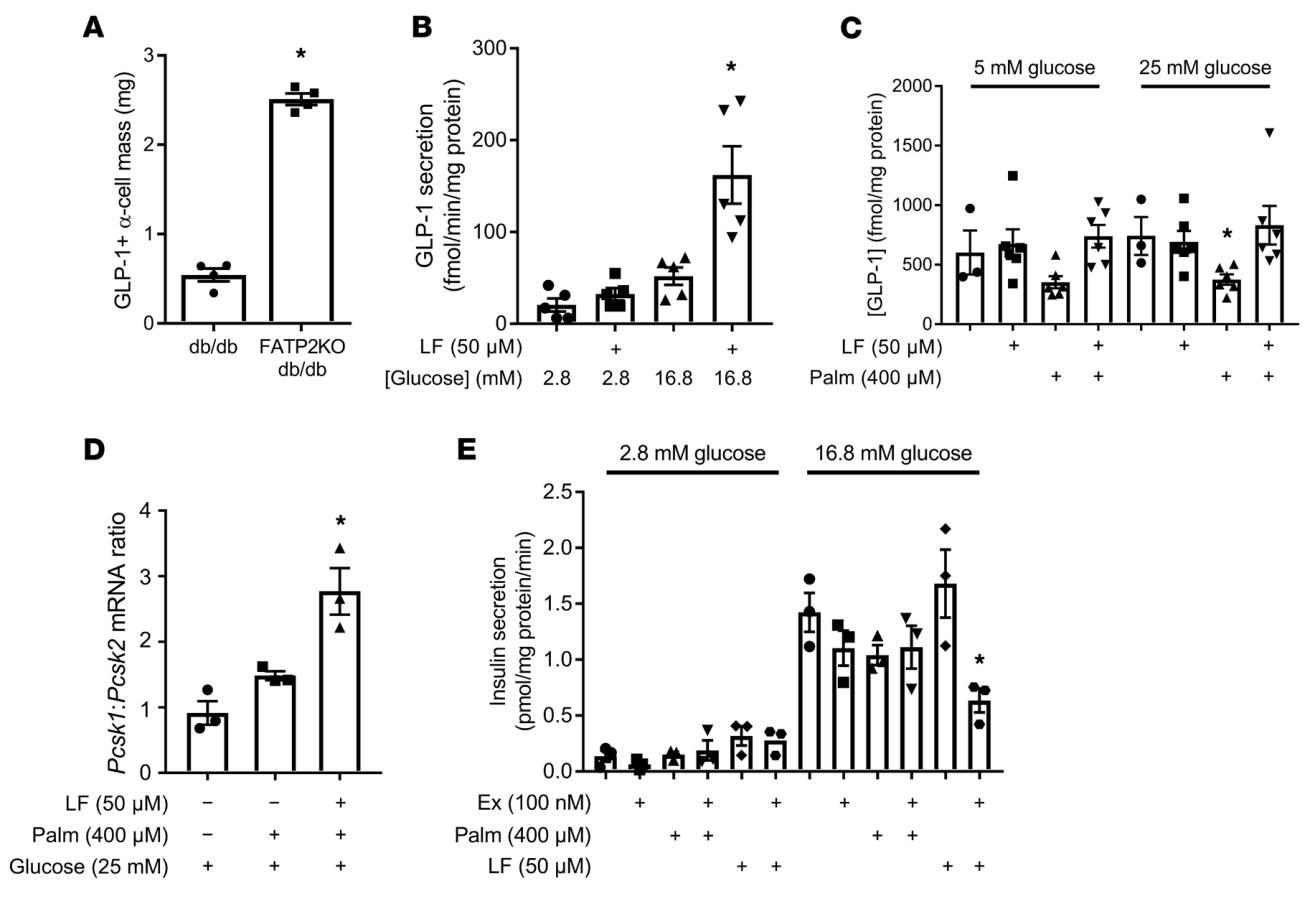
FATP2-KO/inhibition effect on glucose-stimulated GLP-1 and insulin secretion. (**A**) Pancreatic GLP-1^+^ α cell mass was determined as described in Methods in db/db and FATP2-KO db/db mice. *P < 0.01 compared with the db/db group by *t* test. (**B**) Human islets were preincubated with or without lipofermata (LF) and then tested for glucose-stimulated GLP-1 secretion as described in Methods. *P < 0.01 compared with all other groups by ANOVA. (**C**) αTC1-6 cells were preincubated with or without lipofermata or palmitate (Palm) as indicated. Glucose-stimulated GLP-1 secretion was then measured as described in Methods. Each symbol in the scatter bars in **B** and **C** represents 1 sample that was assayed in duplicate (*n* = 3–6 samples per condition). *P < 0.05 compared lipofermata plus palmitate by ANOVA. (**D**) αTC1-6 cells coincubated in 5 mM or 25 mM glucose with or without 400 μM palmitate with or without 50 μM lipofermata for 16 hours were analyzed for *Pcsk1* and *Pcsk2* mRNA expression by qPCR. The results are expressed as the ratio relative to the 5 mM glucose-only condition. *P < 0.05 compared with other groups by ANOVA. (**E**) Glucose-stimulated insulin secretion was measured in human islets, which were preincubated with or without lipofermata and then exposed to exendin[9-39] (Ex) or palmitate, as described in Methods. Each symbol in the scatter bars represents 1 sample that was assayed in duplicate (*n* = 3 samples per condition). *P < 0.01 compared with 16.8 mM glucose plus the lipofermata group by ANOVA. Data represent the mean ± SEM.

The next set of experiments tested the direct effects of FATP2 inhibition on α cell GLP-1 secretion. Both α cell mass and the percentage of GLP-1^+^ α cells were increased in FATP2-KO db/db islets (Supplemental Figure 7). Consequently, the product of the α cell mass and the percentage of GLP-1^+^ α cells (GLP-1^+^ α cell mass) was markedly greater in FATP2-KO db/db mice than in db/db mice (Figure 8A). FATP2 inhibition in human islets enhanced glucose-stimulated GLP-1 secretion, particularly under high-glucose conditions (Figure 8B). Prolonged high glucose plus palmitate has previously been shown to inhibit GLP-1 secretion due to glucolipotoxicity (36). To assess whether FATP2 inhibition preserves GLP-1 secretion, glucose-stimulated GLP-1 release was tested in αTC1-6 cells (37, 38) in response to palmitate with or without lipofermata preincubation (32, 33). Figure 8C demonstrates that under low- and high-glucose conditions, palmitate decreased αTC1-6 cell GLP-1 secretion, which was rescued by lipofermata preincubation. Taken together, the data indicate that FATP2 inhibition or deletion preserved α cell GLP-1 secretion.

Regulation of GLP-1 and glucagon expression is primarily posttranscriptional, with differential cleavage of proglucagon by PC1/3 (encoded by proprotein convertase subtilisin/kexin type 1 [*PCSK1*]) generating GLP-1, and by PC2 (encoded by *PCSK2*) to produce glucagon. Adult α cells express scant PC1/3 and secrete very little basal GLP-1, but in vivo stresses, such as diabetes and aging, cause α cell hyperplasia and a shift from PC2 to PC1/3 expression (39–42). Inhibition of FATP2 was associated with increased expression of *Pcsk1* and an increased ratio of *Pcsk1*/*Pcsk2* mRNA in mouse αTC1-6 cells (Figure 8D).

To investigate whether FATP2 inhibition regulates α cell GLP-1–dependent insulin secretion, GSIS was examined in human islets pretreated with palmitate, lipofermata, and/or the GLP-1 receptor inhibitor exendin[9-39]. Figure 8E demonstrates that lipofermata enhanced insulin secretion (particularly under high-glucose concentration conditions). Importantly, a large proportion of the increase was exendin[9-39] inhibitable, indicating that FATP2 inhibition enhanced α cell secretion of GLP-1, which acts in a paracrine manner to enhance GSIS.

## Discussion

Type 2 diabetes is characterized by initial hyperinsulinemia and subsequent β cell dedifferentiation and insulin deficiency (43). We previously showed that global FATP2 deletion in genetic and inducible mouse models of type 2 diabetes was associated with markedly decreased plasma glucose and increased plasma insulin (11). Using in vivo, ex vivo, and in vitro models, we now show that the mechanism of sustained hyperinsulinemia in the setting of FATP2 inhibition or deletion was α cell–mediated GLP-1 secretion with paracrine stimulation of β cell insulin secretion.

The conventional dogma is that intestinal L cells are the major source of GLP-1, which exerts potent insulinotropic effects. This mechanism has been questioned, however, given the short half-life and potentially insufficient GLP-1 concentration to stimulate GLP-1 receptors on distant β cells. Additionally, deletion of α cell *Pcsk1*, which encodes the enzyme that catalyzes the cleavage of proglucagon to GLP-1, worsens glucose tolerance (39), and overexpression improves glucose tolerance (44). We provide substantial evidence against an enteroendocrine source of GLP-1 as the mechanism for increased plasma insulin in FATP2-KO mice, including (a) no difference between plasma GLP-1 levels or glucose tolerance in db/db FATP2-KO mice following oral versus i.p. glucose loading, (b) nonoverlapping FATP2 and preproglucagon mRNA expression in the intestines, and (c) lack of FATP2 and GLP-1 protein colocalization in intestinal cells. Additional evidence against FATP2-KO regulation of enteroendocrine GLP-1 is the lack of weight loss in diabetic FATP2-KO mice. The mechanism of GLP-1–mediated weight reduction is complex but at least partly involves gut-derived GLP-1 stimulation of the vagus nerve, which leads to anorexia and delayed gastric emptying (45). FATP2-KO db/db mice were slightly heavier than db/db mice, which mitigates against an enteroendocrine GLP-1 mechanism. Furthermore, the lack of weight loss in FATP2-KO db/db mice was accompanied by no difference in food intake (11), which argues against a FATP-KO effect on GLP-1–mediated satiety through stimulation of hypothalamic pro-opiomelanocortin (POMC) neurons. However, db/db mice harbor a leptin receptor mutation, which causes hyperphagia, and may confound the interpretation of GLP-1–regulated satiety and feeding behavior in this mouse model.

Direct evidence to support α cell–mediated GLP-1 secretion as the mechanism of FATP2-KO–associated hyperinsulinemia included (a) colocalization of FATP2 with human and mouse islet α, but not β, cells, (b) FATP2 mRNA expression in human and mouse α, but not β, cells, (c) inhibition of fatty acid uptake by FATP2 inhibitors in α cells, (d) increased GLP-1^+^ α cell mass in FATP2-KO db/db mice, (e) an increased *Pcsk1*/*Pcsk2* mRNA ratio in αTC1-6 cells treated with lipofermata, and (f) enhanced GLP-1 secretion and exendin[9-39]–inhibitable GSIS in FATP2 inhibitor–treated human islets.

The contribution of glucagon to glucose homeostasis in FATP2-KO db/db mice was relatively minor. In the nonfasting, fed state, when β cells are active, plasma glucagon was decreased in FATP2-KO db/db mice compared with db/db mice, suggesting that glucagon was not the insulin stimulus. To address the glucagon effect more carefully, alanine tolerance tests were conducted in mice that were fasting, which maximally stimulates glucagon, but not insulin secretion (46). The blunted effect on glucose in FATP2-KO db/db mice compared with db/db mice indicates that some of the glucose-lowering effect of FATP2 deletion could be due to suppressed glucagon-stimulated hepatic gluconeogenesis. While the relatively modest plasma glucose increases in FATP2-KO db/db mice compared with WT mice could result in some glucagon-stimulated insulin release, synthesis of the data from all metabolic studies most strongly indicate that the predominant FATP2 gene deletion effect on glycemia was the paracrine effect of GLP-1 secretion by α cells.

Prior investigation of fatty acid effects on GLP-1 secretion is limited. Incubation of a fatty acid mixture with αTC1-6 cells stimulates GLP-1 secretion at low glucose and suppresses GLP-1 in high-glucose conditions (36). Similar results were observed in L cells, with palmitate inhibition of GLP-1 secretion (47). The stimulation of GLP-1 is primarily by unsaturated fatty acids (36, 48) and is presumed to be mediated by G protein–coupled FFAR4 in α cells (49) and FATP4 or FFAR1 in L cells (48, 50).

FATP2 inhibition was associated with an increased *Pcsk1*/*Pcsk2* mRNA ratio. These data are consistent with palmitate-induced inhibition of islet and L cell PC1/3 (51–53), which catalyzes the conversion of proglucagon to GLP-1 and of proinsulin to insulin (54). Although previous islet studies focused on PC1/3 effects on proinsulin cleavage in β cells (51–53), it is plausible that FATP2 inhibition also enhances GLP-1 by blocking palmitate-induced PC1/3 suppression in α cells. Future studies will be required to explore other potential mechanisms of enhanced α cell GLP-1 secretion, including inhibition of lipotoxicity (36, 47) and reciprocal stimulation of GLP-1 by insulin. While a feed-forward (insulin-stimulating GLP-1) mechanism has been proposed for intestinal L cells (55), similar results in α cells have not been described, to our knowledge.

The insulinotropic effect of GLP-1 on β cells is due primarily to GLP-1 receptor–mediated augmentation of GSIS. However, sustained hyperinsulinemia in FATP2-KO db/db mice was also facilitated by increased β cell mass and islet hypertrophy, which is a predominately GLP-1–independent process. GLP-1 exerts cytoprotective effects in β cell lines (56, 57), but in human islets GLP-1 does not stimulate β cell mitogenesis or affect islet size (57, 58). Identification of the FATP2-KO–regulated factors that stimulate islet hypertrophy will require further investigation.

The effects of fatty acids on insulin secretion depend on many factors, including fatty acid carbon chain length and saturation, the chronicity of exposure, the fasting state, and the concomitant glucose concentration (16, 59). While palmitate suppressed GLP-1 secretion in αTC1-6 cells, we observed a more modest effect of palmitate on insulin secretion in human islets. However, even in the absence of fatty acids, GSIS was enhanced in lipofermata-treated islets, suggesting that FATP2 inhibition may mediate insulinotropic effects independent of fatty acid uptake. To date, FATP2 has been linked primarily to lipid metabolism, particularly PPARα-regulated genes (6), which have no effect on pancreatic insulin release (60). Downstream pathways from FATP2 are largely unexplored, and identification of additional FATP2-directed events, which may regulate GLP-1–dependent GSIS, are therefore warranted.

We conclude that FATP2 deletion or inhibition exerts glucose-lowering effects through α cell–mediated GLP-1 secretion and paracrine β cell insulin release. One potential clinical implication is that, in contrast to diabetes treatment with GLP-1 receptor agonists, which ostensibly mimic the effect of endogenous GLP-1, FATP2 inhibition may represent a more natural stimulus of α cell GLP-1 augmentation. Moreover, FATP2 inhibition could represent a potential adjunctive glucose-lowering therapy and/or a means to delay the onset of type 2 diabetes.

## Methods

### Sex as a biological variable.

With the exception of high-fat diet experiments, in which only male mice develop obesity, no differences were noted between male and female mice for any parameters. Therefore, equal numbers of male and female mice were used in all other experiments.

### Mice.

Conditional proximal tubule FATP2-KO mice were generated from intercrosses between *GGT1*-Cre (The Jackson Laboratory) and *Slc27a2*-floxed mice (gift from Dmitry Gabrilovich, The Wistar Institute, Philadelphia, Pennsylvania, USA [ref. 61]) on a congenic C57BL-KS/J background. Genotyping by PCR from toe samples was done by Transnetyx. Type 2 diabetes was induced as previously described (11). Briefly, at 6 weeks of age, male mice were fed a high-fat diet (Harlan, Teklad TD.06414, 60.3% fat, 21.3% carbohydrate, 18.4% protein) for 6 months. After 3 months of a high-fat diet, mice were administered low-dose i.p. streptozotocin (45 μg/g) daily for 3 consecutive days. Diabetes was defined by fasting glucose of greater than 200 mg/dL. Glucose and GLP-1 were assayed after fasting from 6 am to 10 am. Tail vein blood glucose was assayed by glucometer, as previously described (11).

### Immunohistochemistry.

Paraffin-embedded human ileum and duodenum slides were purchased from Zyagen. Deparaffinized sections were treated with sodium citrate (10 mM, pH 6.0) for antigen retrieval. Mouse pancreas samples were fixed in paraformaldehyde (4%, 24 hours, room temperature), cryopreserved in sucrose (30% in PBS overnight), and frozen at –80^o^ C. Frozen sections (5 μm by cryostat) were permeabilized with Triton X-100 (MilliporeSigma; 0.2% in PBS, 10 minutes, room temperature) and blocked with donkey serum (5% in PBS, 1 hour, room temperature). The primary antibodies are listed in Supplemental Table 1. Sections were mounted in SlowFade Diamond Antifade Mountant with DAPI (Invitrogen, Thermo Fisher Scientific) and viewed with a Leica or Olympus confocal microscope.

### β cell and GLP-1^+^ α cell mass.

Calculation of α and β cell mass was done using published methods (62). Mouse pancreas tissue was fixed in paraformaldehyde (4%, 24 hours, room temperature), weighed, cryopreserved in sucrose (30% in PBS overnight), and frozen at –80°C. Three 5 μm frozen sections, cut 200 μm apart, were analyzed. We labeled α and β cells with glucagon and insulin antibodies, respectively, as previously described (11). Areas of α and β cells were normalized to total pancreas area, and these values were then multiplied by the pancreas weight to obtain α and β cell mass. GLP-1^+^ α cell mass was determined by multiplying the α cell mass and the percentage of glucagon^+^ cells that colabeled with GLP-1 antibodies.

### Reverse transcriptase PCR.

The methods for reverse transcriptase PCR (RT-PCR) have previously been described in detail (11). Briefly, total RNA was extracted from whole mouse organs, the mouse αTC1-6 pancreatic α cell line (ATCC), or the rat INS-1 pancreatic β cell line (gift from Yisheng Yang, Case Western Reserve University, Cleveland, Ohio, USA) using the RNeasy Mini Kit (Qiagen). RNA concentrations were determined using the NanoDrop 2000 Spectrophotometer (Thermo Fisher Scientific). Reverse transcription was performed using 5 μg total RNA, and cDNA was generated using the SuperScript III First-Strand Synthesis System (Invitrogen, Thermo Fisher Scientific). Human α cell cDNA was purchased from Celprogen. PCR reactions from 1.5 μg cDNA were conducted in 20 μL volume using EmeraldAmp Max PCR Master Mix 2X Premix (Takara Bio), according to the recommended protocol and PCR cycling conditions, for 30 cycles (35 cycles for INS-1 cell PCR). Primers were purchased from Eurofins Genomics, and the sequences are listed in Supplemental Table 2. PCR products underwent 2% agarose gel electrophoresis, and bands were identified by ethidium bromide (Invitrogen, Thermo Fisher Scientific) staining and then photographed. qPCR was conducted as previously described (63). Briefly, cDNA was generated using the SuperScript III First-Strand System and amplified using Radiant SYBR Green 2x Master Mix (Alkali Scientific) and the QuantStudio 3 System (Applied Biosystems). Quantification was determined by the comparative Ct (ΔΔCt) method.

### Fatty acid uptake in αTC1-6 cells.

Experiments were conducted according to previously described methods (20), using a QBT assay (Molecular Devices). Briefly, αTC1-6 cells (American Type Culture Collection [ATCC]), originally derived from mouse pancreatic α cells (64), were seeded in 96-well plates and cultured to confluence over 24 hours. Wells were washed with serum-free, phenol-free media for 2 hours at 37° C. Lipofermata (5-bromo-5′-phenylspiro[3*H*-1,3,4-thiadiazole-2,3′-indoline]-2-1) (MedChemExpress) was robotically incubated for the final hour. BODIPY-conjugated C18 fatty acids (Molecular Devices QBT assay, 2.5 μM complexed with 0.2 % fatty acid-free albumin carrier + lipofermata in QBT loading buffer that contains a proprietary external quenching dye) were robotically added at time = 0. Excitation at λ = 490 nm pulses were delivered, and emission at λ = 510 nm was recorded at 15-second intervals for 10 minutes. BODIPY-labeled fatty acid uptake was determined from fluorescence values obtained at 60 seconds, which is the static time point that most highly correlated with maximum velocity (Supplemental Figure 8). Plates were imaged on the Synergy Neo2 HTX Multi-Mode Microplate reader (BioTek) and averaged from 6 fields captured from each well using Gen5 software. IC_50_ values were calculated using GraphPad Prism 7 software.

### Hormone assays.

Glucagon (10-I271-01), GLP-1 (10-I278-01), and human insulin (10-1113-01), were assayed from mouse plasma or culture media by ELISA (all from Mercodia).

### In vivo metabolic analyses.

To facilitate multiple blood samples for GLP-1 assays, a carotid artery catheter was placed the day prior to experiments. Mice were fasted for 4 hours (6 am to 10 am) prior to the OGTT (2 g/kg by gavage) or IPGTT (2 g/kg i.p.) (65). Arterial blood was drawn at baseline and then 1 hour after glucose administration, and plasma was saved at –80°C in tubes containing linagliptin (MedChemExpress; 100 nM) for GLP-1 assays later. Glucometer readings were obtained at baseline, 30, 60, 90 and 120 minutes. For alanine tolerance tests, fasted mice were administered L-alanine (2 g/kg i.p.), and glucometer readings were obtained at baseline, 10, 30, 45, 60 and 120 minutes. Nonfasting blood samples for glucagon were obtained from mice by cardiac puncture at the time of sacrifice.

### Glucose-stimulated GLP-1 and insulin secretion.

Human islets (ProdoLabs) were cultured according to established methods (66). Islets were identified under a dissecting microscope and suspended in RPMI 1640 plus 15% FBS for at least 16 hours prior to experimentation. Islets were equilibrated in Petri dishes containing modified Krebs buffer (2 mM NaHCO_3_, 10 mM HEPES, pH 7.4, 37°C, 1 hour). Ten islets were selected and deposited in cell culture wells, with quintuplicate wells for each condition. Initial incubations included modified Krebs buffer supplemented with 2.8 mM glucose (37°C, 1 hour) plus linagliptin (MedChemExpress; 100 nM) with or without palmitate (Avanti; 100–400 μM complexed with 0.2% delipidated albumin), lipofermata (MedChemExpress; 50 μM), or exendin[9-39] (MedChemExpress; 100 nM). After 1 hour, minimum volumes of media for GLP-1 or insulin ELISA assays in duplicate were saved at –80°C. Identical conditions were then repeated for an additional hour in Krebs buffer with high glucose (16.8 mM, 37°C, 1 hour). Islets were pelleted by centrifugation, lysed in SDS-PAGE buffer, and assayed for protein content (Nanodrop; absorption at λ = 280 nm). A similar protocol was followed for glucose-stimulated GLP-1 secretion in αTC1-6 cells, except for incubations in 5 mM and 25 mM glucose, to conform with established methods for these cells (37, 38).

### Data availability.

Data in the manuscript are available in the Supporting Data Values file.

### Statistics.

Graphical data are presented as the mean ± SEM and were analyzed using GraphPad Prism 7 (GraphPad Software). Data from multiple groups were analyzed by 1-way ANOVA and Tukey’s post hoc test for multiple comparisons. Data from 2 groups were analyzed by unpaired, 2-tailed *t* test. Statistical significance for all analyses is defined as a *P* value of less than 0.05.

### Study approval.

All studies were conducted in accordance with protocols approved by the IACUC of Case Western Reserve University School of Medicine.

## Author contributions

SK, RJG, ZL, and VL conducted experiments, acquired data, analyzed data, and reviewed and edited the manuscript. IS contributed reagents, contributed to discussions, and reviewed and edited the manuscript. JS contributed to discussions and reviewed and edited the manuscript. POO conducted experiments, acquired data, and reviewed and edited the manuscript. JLG conducted experiments, analyzed data, and reviewed and edited the manuscript. DA designed research studies, analyzed data, and reviewed and edited the manuscript. JRS obtained funding, designed research studies, analyzed data, and wrote and edited the manuscript.

## Funding support

This work is the result of NIH funding, in whole or in part, and is subject to the NIH Public Access Policy. Through acceptance of this federal funding, the NIH has been given a right to make the work publicly available in PubMed Central.

- NIH grant 5R01DK064819 (to DA).
- NIH grant 2R01DK067528 (to JRS).

## Supplementary Material

Supplemental data

Unedited blot and gel images

Supporting data values

## Version 1 09/16/2025

In-Press Preview

## Version 2 12/01/2025

Electronic publication

## References

[1] Reidy K (2014). Molecular mechanisms of diabetic kidney disease. J Clin Invest.

[2] Eid S (2019). New insights into the mechanisms of diabetic complications: role of lipids and lipid metabolism. Diabetologia.

[3] Sobczak AIS (2019). Changes in plasma free fatty acids associated with type-2 diabetes. Nutrients.

[4] Hirsch D (1998). A family of fatty acid transporters conserved from mycobacterium to man. Proc Natl Acad Sci U S A.

[5] Qiu P (2020). FATP2-targeted therapies - A role beyond fatty liver disease. Pharmacol Res.

[6] Perez VM (2020). Deletion of fatty acid transport protein 2 (FATP2) in the mouse liver changes the metabolic landscape by increasing the expression of PPARα-regulated genes. J Biol Chem.

[7] Falcon A (2010). FATP2 is a hepatic fatty acid transporter and peroxisomal very long-chain acyl-CoA synthetase. Am J Physiol Endocrinol Metab.

[8] Lewis DL, Wolff JA (2005). Delivery of siRNA and siRNA expression constructs to adult mammals by hydrodynamic intravascular injection. Methods Enzymol.

[9] Bradley SP (2005). Gene silencing in the endocrine pancreas mediated by short-interfering RNA. Pancreas.

[10] Larson SD (2007). Effectiveness of siRNA uptake in target tissues by various delivery methods. Surgery.

[11] Khan S (2020). Fatty acid transport protein-2 regulates glycemic control and diabetic kidney disease progression. JCI Insight.

[12] Holter MM (2022). Alpha cell paracrine signaling in the regulation of beta cell insulin secretion. Front Endocrinol.

[13] Jorsal T (2018). Enteroendocrine K and L cells in healthy and type 2 diabetic individuals. Diabetologia.

[14] Mancini AD, Poitout V (2013). The fatty acid receptor FFA1/GPR40 a decade later: how much do we know?. Trends Endocrinol Metab.

[15] Campbell JE, Newgard CB (2021). Mechanisms controlling pancreatic islet cell function in insulin secretion. Nat Rev Mol Cell Biol.

[16] Haber EP (2006). New insights into fatty acid modulation of pancreatic beta cell function. Int Rev Cytol.

[17] Marchetti P (2012). A local glucagon-like peptide 1 (GLP-1) system in human pancreatic islets. Diabetologia.

[18] Ellingsgaard H (2011). Interleukin-6 enhances insulin secretion by increasing glucagon-like peptide-1 secretion from L cells and alpha cells. Nat Med.

[19] Kieffer TJ (1995). Degradation of glucose-dependent insulinotropic polypeptide and truncated glucagon-like peptide 1 in vitro and in vivo by dipeptidyl peptidase IV. Endocrinology.

[20] Khan S (2018). Kidney proximal tubule lipoapoptosis is regulated by fatty acid transporter-2 (FATP2). J Am Soc Nephrol.

[21] Park J (2018). Single cell transcriptomics of the mouse kidney reveals potential cellular targets of kidney disease. Science.

[22] Gerich JE (2010). Role of the kidney in normal glucose homeostasis and in the hyperglycaemia of diabetes mellitus: therapeutic implications. Diabet Med.

[23] Schrimpe-Rutledge AC (2012). Discovery of novel glucose-regulated proteins in isolated human pancreatic islets using LC-MS/MS-based proteomics. J Proteome Res.

[24] Dalbøge LS (2013). Characterisation of age-dependent beta cell dynamics in the male db/db mice. PLoS One.

[25] Adriaenssens AE (2016). Transcriptomic profiling of pancreatic alpha, beta and delta cell populations identifies delta cells as a principal target for ghrelin in mouse islets. Diabetologia.

[26] Muraro MJ (2016). A single cell transcriptome atlas of the human pancreas. Cell Syst.

[27] Segerstolpe Å (2016). Single cell transcriptome profiling of human pancreatic islets in health and type 2 diabetes. Cell Metab.

[28] Tarifeño-Saldivia E (2017). Transcriptome analysis of pancreatic cells across distant species highlights novel important regulator genes. BMC Biol.

[29] Oropeza D (2021). Stage-specific transcriptomic changes in pancreatic α cells after massive β cell loss. BMC Genomics.

[30] Sturgill D (2024). PancrESS - a meta-analysis resource for understanding cell-type specific expression in the human pancreas. BMC Genomics.

[31] Melton EM (2011). Human fatty acid transport protein 2a/very long chain acyl-CoA synthetase 1 (FATP2a/Acsvl1) has a preference in mediating the channeling of exogenous n-3 fatty acids into phosphatidylinositol. J Biol Chem.

[32] Ahowesso C (2015). Chemical inhibition of fatty acid absorption and cellular uptake limits lipotoxic cell death. Biochem Pharmacol.

[33] Kumar M (2023). Definition of fatty acid transport protein-2 (FATP2) structure facilitates identification of small molecule inhibitors for the treatment of diabetic complications. Int J Biol Macromol.

[34] Weir GC, Bonner-Weir S (2023). Conflicting views about interactions between pancreatic α cells and β cells. Diabetes.

[35] Small L (2022). Comparative analysis of oral and intraperitoneal glucose tolerance tests in mice. Mol Metab.

[36] Sancho V (2017). Metabolic regulation of GLP-1 and PC1/3 in pancreatic α cell line. PLoS One.

[37] Whalley NM (2011). Processing of proglucagon to GLP-1 in pancreatic α cells: is this a paracrine mechanism enabling GLP-1 to act on β cells?. J Endocrinol.

[38] Piro S (2014). Chronic exposure to GLP-1 increases GLP-1 synthesis and release in a pancreatic alpha cell line (α-TC1): evidence of a direct effect of GLP-1 on pancreatic alpha cells. PLoS One.

[39] Traub S (2017). Pancreatic α cell-derived glucagon-related peptides are required for β cell adaptation and glucose homeostasis. Cell Rep.

[40] Kilimnik G (2010). Intraislet production of GLP-1 by activation of prohormone convertase 1/3 in pancreatic α cells in mouse models of β cell regeneration. Islets.

[41] O’Malley TJ (2014). Progressive change of intra-islet GLP-1 production during diabetes development. Diabetes Metab Res Rev.

[42] Hansen AM (2011). Upregulation of alpha cell glucagon-like peptide 1 (GLP-1) in Psammomys obesus—an adaptive response to hyperglycaemia?. Diabetologia.

[43] Talchai C (2012). Pancreatic β cell dedifferentiation as a mechanism of diabetic β cell failure. Cell.

[44] Wideman RD (2006). Improving function and survival of pancreatic islets by endogenous production of glucagon-like peptide 1 (GLP-1). Proc Natl Acad Sci U S A.

[45] Kanoski SE (2016). GLP-1 and weight loss: unraveling the diverse neural circuitry. Am J Physiol Regul Integr Comp Physiol.

[46] Capozzi ME (2019). Glucagon lowers glycemia when β cells are active. JCI Insight.

[47] Filippello A (2018). Chronic exposure to palmitate impairs insulin signaling in an intestinal L cell line: a possible shift from GLP-1 to glucagon production. Int J Mol Sci.

[48] Thombare K (2017). Long chain saturated and unsaturated fatty acids exert opposing effects on viability and function of GLP-1-producing cells: Mechanisms of lipotoxicity. PLoS One.

[49] Campbell SA (2021). Evidence for the existence and potential roles of intra-islet glucagon-like peptide-1. Islets.

[50] Poreba MA (2012). Role of fatty acid transport protein 4 in oleic acid-induced glucagon-like peptide-1 secretion from murine intestinal L cells. Am J Physiol Endocrinol Metab.

[51] Wen Q (2023). Metformin restores prohormone processing enzymes and normalizes aberrations in secretion of proinsulin and insulin in palmitate-exposed human islets. Diabetes Obes Metab.

[52] Iida H (2023). SERCA2 regulates proinsulin processing and processing enzyme maturation in pancreatic beta cells. Diabetologia.

[53] Hayashi H (2014). Glucagon-like peptide-1 production in the GLUTag cell line is impaired by free fatty acids via endoplasmic reticulum stress. Metabolism.

[54] Ramzy A (2020). Revisiting proinsulin processing: evidence that human β cells process proinsulin with prohormone convertase (PC) 1/3 but not PC2. Diabetes.

[55] Yi F (2008). Cross talk between the insulin and Wnt signaling pathways: evidence from intestinal endocrine L cells. Endocrinology.

[56] Cornu M (2009). Glucagon-like peptide-1 protects beta cells against apoptosis by increasing the activity of an IGF-2/IGF-1 receptor autocrine loop. Diabetes.

[57] Drucker DJ (2013). Incretin action in the pancreas: potential promise, possible perils, and pathological pitfalls. Diabetes.

[58] Rosselot C (2024). Harmine and exendin-4 combination therapy safely expands human β cell mass in vivo in a mouse xenograft system. Sci Transl Med.

[59] Prentki M (2013). Metabolic signaling in fuel-induced insulin secretion. Cell Metab.

[60] Guerre-Millo M (2001). PPAR-alpha-null mice are protected from high-fat diet-induced insulin resistance. Diabetes.

[61] Veglia F (2019). Fatty acid transport protein 2 reprograms neutrophils in cancer. Nature.

[62] Bru-Tari E (2019). Pancreatic alpha cell mass in the early-onset and advanced stage of a mouse model of experimental autoimmune diabetes. Sci Rep.

[63] Lakhe-Reddy S (2006). Beta8 integrin binds Rho GDP dissociation inhibitor-1 and activates Rac1 to inhibit mesangial cell myofibroblast differentiation. J Biol Chem.

[64] Powers AC (1990). Proglucagon processing similar to normal islets in pancreatic alpha-like cell line derived from transgenic mouse tumor. Diabetes.

[65] Al Rijjal D, Wheeler MB (2022). A protocol for studying glucose homeostasis and islet function in mice. STAR Protoc.

[66] Son J (2023). Genetic and pharmacologic inhibition of ALDH1A3 as a treatment of β cell failure. Nat Commun.

