# Supplementary material for: Fatty acid transport protein 2 inhibition enhances glucose tolerance through α cell–mediated GLP-1 secretion
Source: J Clin Invest. 2025 Sep 16;135(23):e192011. doi: 10.1172/JCI192011 (PMC12646670; doi:10.1172/JCI192011)
Supplement: Supplemental data [file jci-135-192011-s216.pdf]

Supplemental Table 1. Primary antibodies and conditions

AF488-conjugated rabbit anti–insulin IgG (9016, Cell Signaling; 1:200, 16 hours, 4°C)
eFluor 570-conjugated mouse monoclonal anti–glucagon IgG (ICACLS, Invitrogen; 10 µg/mL, 16 hours, 4°C)
eFluor 660-conjugated mouse anti-somatostatin (ICDCLS, Invitrogen; 5 µg/mL, 16 hours, 4°C)
Rabbit anti–FATP2 IgG (GTX115526, GeneTex; 1:50, 16 hours, 4°C)
Mouse anti-human GLP-1 (8G, Invitrogen; 1:200, 16 hours, 4°C)
Rabbit anti-GLP-1 IgG (G-028-13, Phoenix; 1:200, 16 hours, 4°C)

Supplemental Table 2. PCR primers

Figure 3A

h/mFATP2_Forward: GGAGATACATTCCGGTGGAA
h/mFATP2_Reverse: TGATCTCAATGGTGTCCTGT
mGAPDH_Forward: CTGCCATTTGCAGTGGCAAAGTGG
mGAPDH_Reverse: TTGTCATGGATGACCTTGGCCAGG
hGAPDH_Forward: GTCTTCACCACCATGGAGAAG
hGAPDH_Reverse: GCTTCACCACCTTCTTGATGTCATC

Figure 3B

mFATP2_Forward: TGTGCCAGGTCATGAGGGTCG
mFATP2_Reverse: CTCAATGGTATCTTGTATCCTCAGGAA
mGAPDH_Forward: GTGGAAGGGCTCATGACCACAG
mGAPDH_Reverse: ATACTTGGCAGGTTTCTCCAGGC

Figures 5A and 5B

mFATP2_Forward: TGTGCCAGGTCATGAGGGTCG
mFATP2_Reverse: CTCAATGGTATCTTGTATCCTCAGGAA
mGCG_Forward: CCTTCAAGACACAGAGGAGAACC
mGCG_Reverse: CTGTAGTCGCTGGTGAATGTGC
mGAPDH_SYBR_Forward: GTGGAAGGGCTCATGACCACAG
mGAPDH_SYBR_Reverse: CCCGTTCAGCTCTGGGATGAC

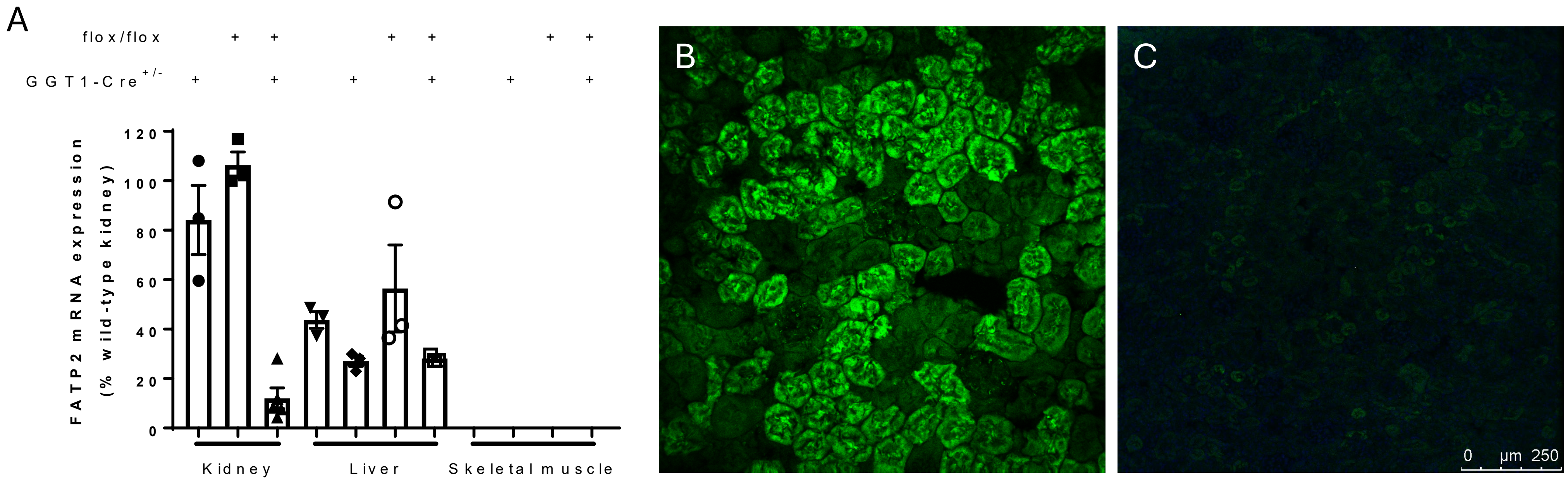

Supplemental Figure 1. qPCR for Slc27a2 mRNA expression in tissues with designated genotypes (A). Data are means ± SEM from three mice, and values are normalized to wild-type kidney expression, which is defined as 100. Immunohistochemical labeling of FATP2 in renal cortex from flox/flox (B) and GGT1-Cre$^{+/-}$ flox/flox (C) mice.

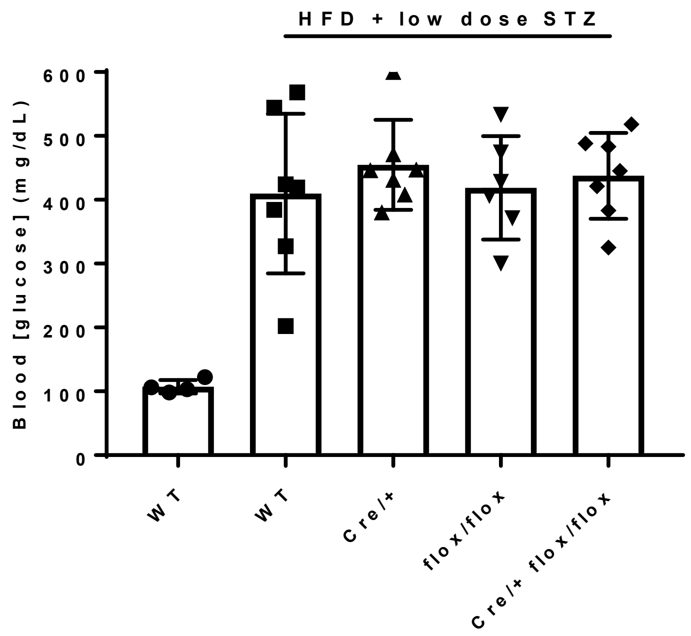

Supplemental Figure 2. Blood glucose in mice with proximal tubule FATP2 gene deletion. Six months old C57BLKS/J wild-type (WT), GGT1-Cre-lox, GGT1-Cre and floxed controls with diabetes induced by high fat diet (HFD) and streptozotocin (STZ) (see Methods for details) were fasted for four hours, and tail vein blood glucose was measured by glucometer. Blood glucose was normal for all genotypes in the absence of HFD and STZ (not shown).

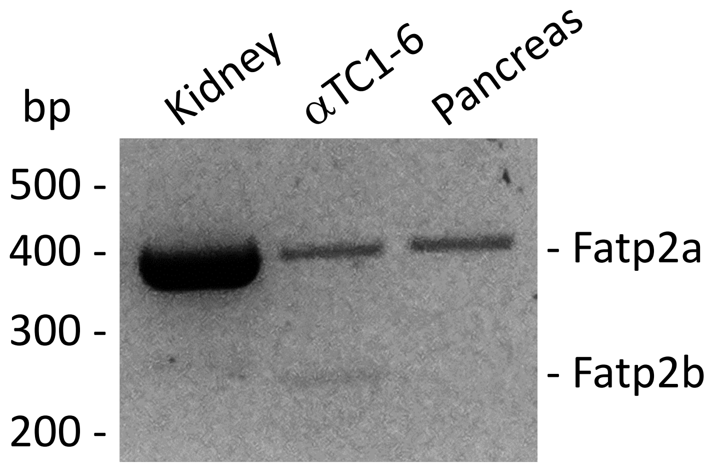

Supplemental Figure 3. RNA extracted from mouse kidney (positive control), αTC1-6 cells and mouse pancreas was amplified by RT-PCR using mouse primers that flank exon 3. The 401 bp band corresponds to full-length Fatp2a, and the 242 bp pair band corresponds to Fatp2b, which encodes the Fatp2 isoform that lacks intrinsic acyl co-A synthetase activity.

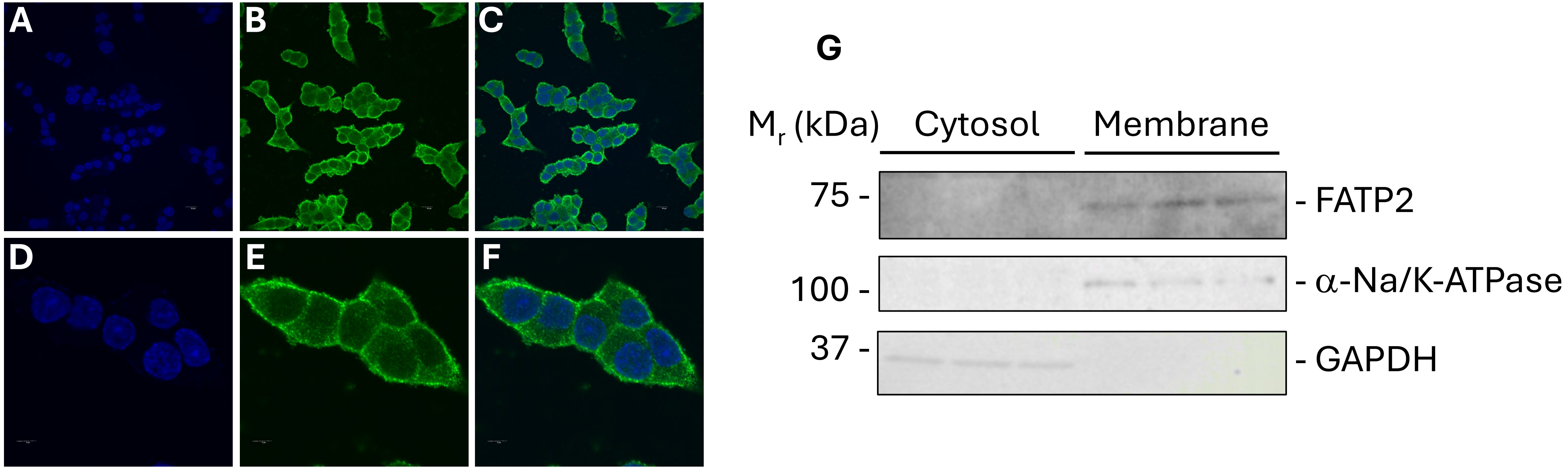

Supplemental Figure 4. αTC1-6 cells were fixed and immunostained for FATP2 as described in Methods. A-C, 200X magnification; D-F, 600X magnification. A and D, DAPI nuclear stain; B and E, FATP2 expression; C and F, merged images. G, immunoblots of αTC1-6 cytosol and membrane fractions, which were probed for FATP2 (Abcam, ab228784), and then stripped and re-probed for the α-subunit of Na/K-ATPase (Upstate Biotechnology, #05-369) and GAPDH (Cell Signaling, #2118), as markers of membrane and cytosol, respectively. Each lane represents a separate cell lysate collection.

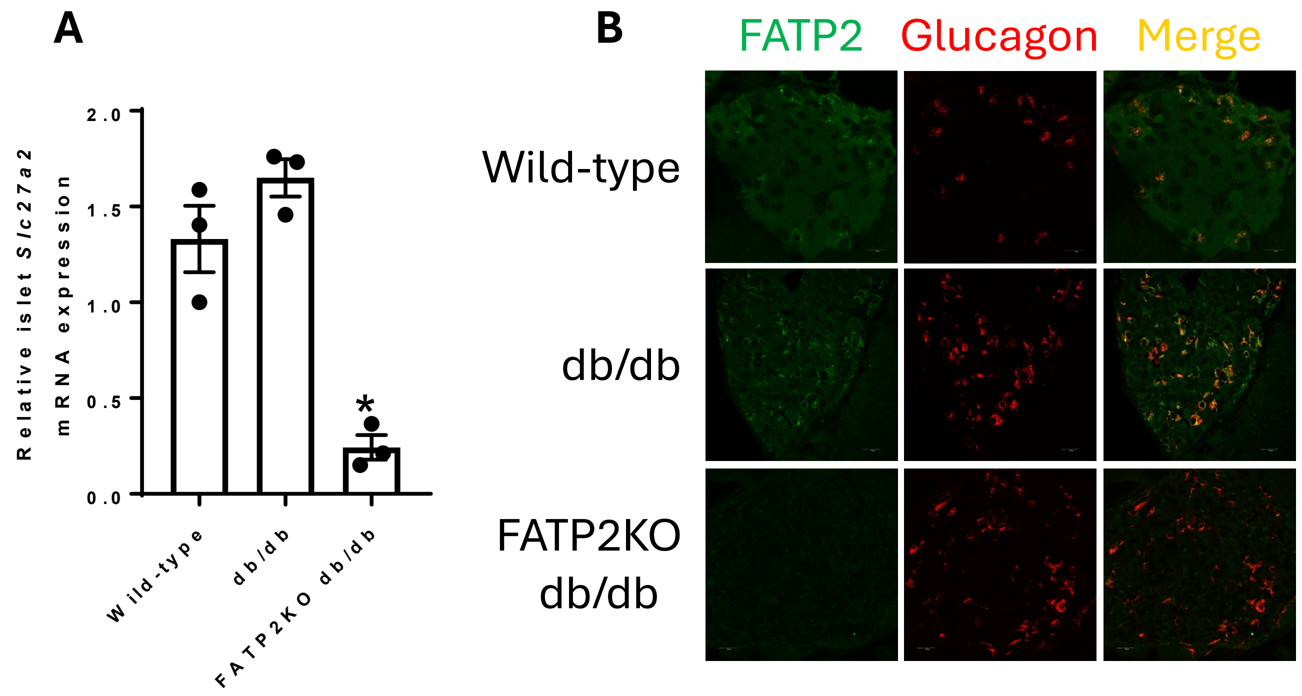

Supplemental Figure 5. **A**, Total RNA was extracted from mouse islets, which were isolated according to previously described methods (66). Slc27a2 mRNA expression from wild-type, db/db and FATP2KO db/db mice was determined in triplicate by qPCR. Data are normalized to islet Slc27a2 mRNA content in wild-type mice from first experiment and expressed as mean ± SEM. * P <0.05 between groups by ANOVA. **B**, Immunohistochemical labeling of FATP2 and glucagon in islets from wild-type, db/db and FATP2KO db/db mice.

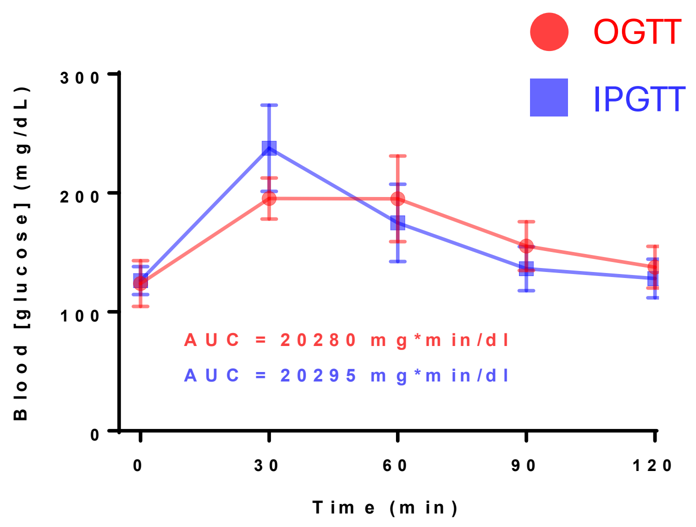

Supplemental Figure 6. OGTT and IPGTT were conducted in FATP2KO mice, as described in Methods. Blood glucose was determined at the indicated times in six mice per group.

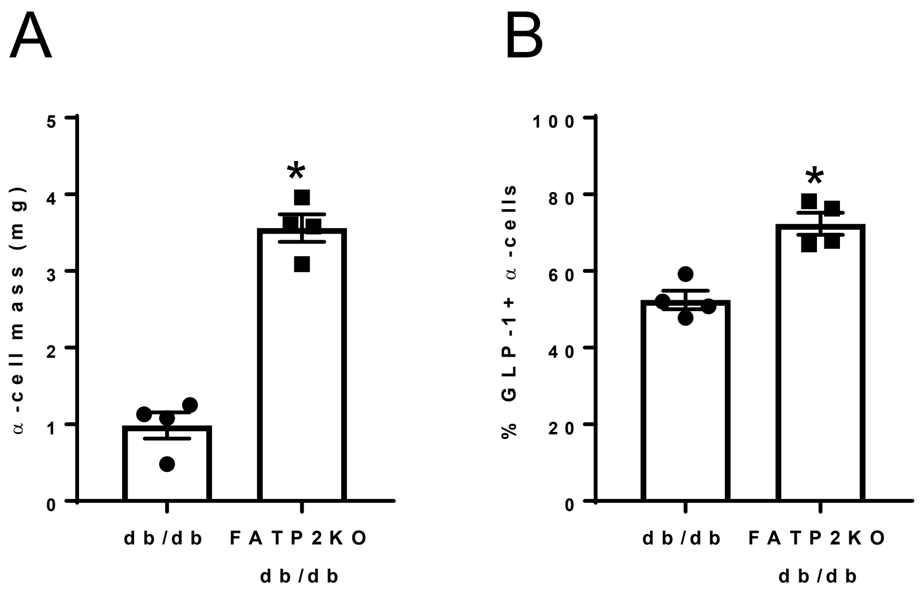

Supplemental Figure 7. α-cell mass (A) and % GLP-1+ α-cells (B) were determined as described in Methods in islets from db/db and FATP2KO db/db mice. Each symbol in the scatter bars represents one experiment. Therefore, N = 4 experiments per condition. * P <0.01 compared to db/db group.

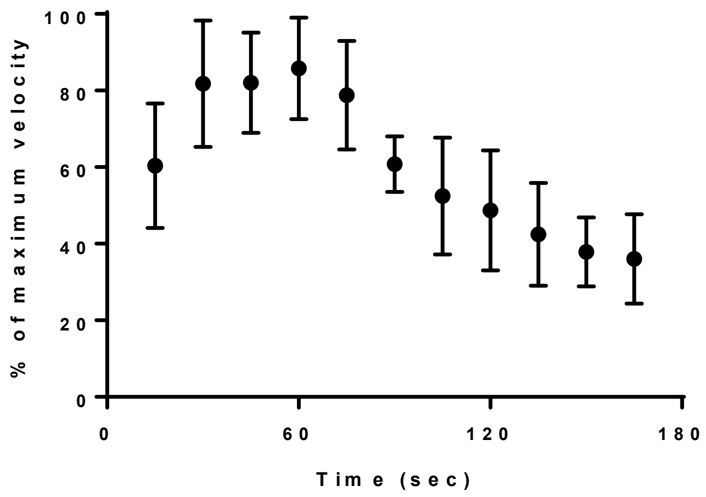

Supplemental Figure 8. BODIPY-labeled fatty acid uptake was measured as described in Methods. Fluorescence emission was determined at 15 sec intervals, and a tangent corresponding to the time point with the greatest slope was defined as maximum (100%) velocity. The ratio of the static fluorescence value at each time point/the fluorescence value corresponding to maximum velocity was then expressed as the % of maximum velocity. Data are mean ± SEM from four experiments.
