# Supplementary material for: Fatty acid transport protein 2 inhibition enhances glucose tolerance through α cell–mediated GLP-1 secretion
Source: J Clin Invest. 2025 Sep 16;135(23):e192011. doi: 10.1172/JCI192011 (PMC12646670; doi:10.1172/JCI192011)

Full unedited blot for Supplemental Figure 4B

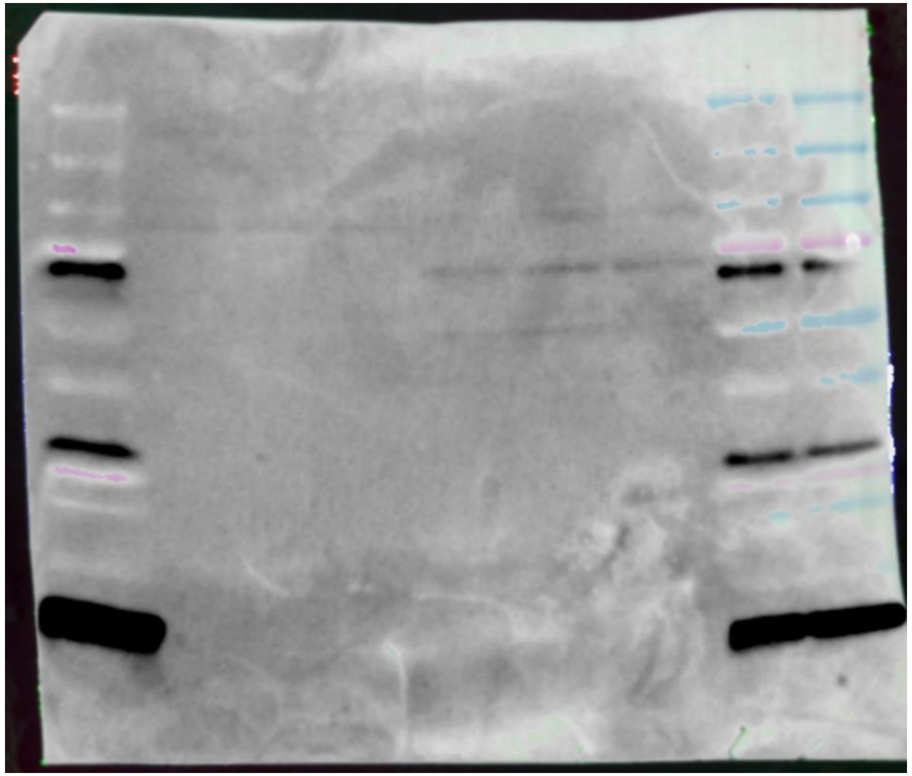

- FATP2

Full unedited blot for Supplemental Figure 4B

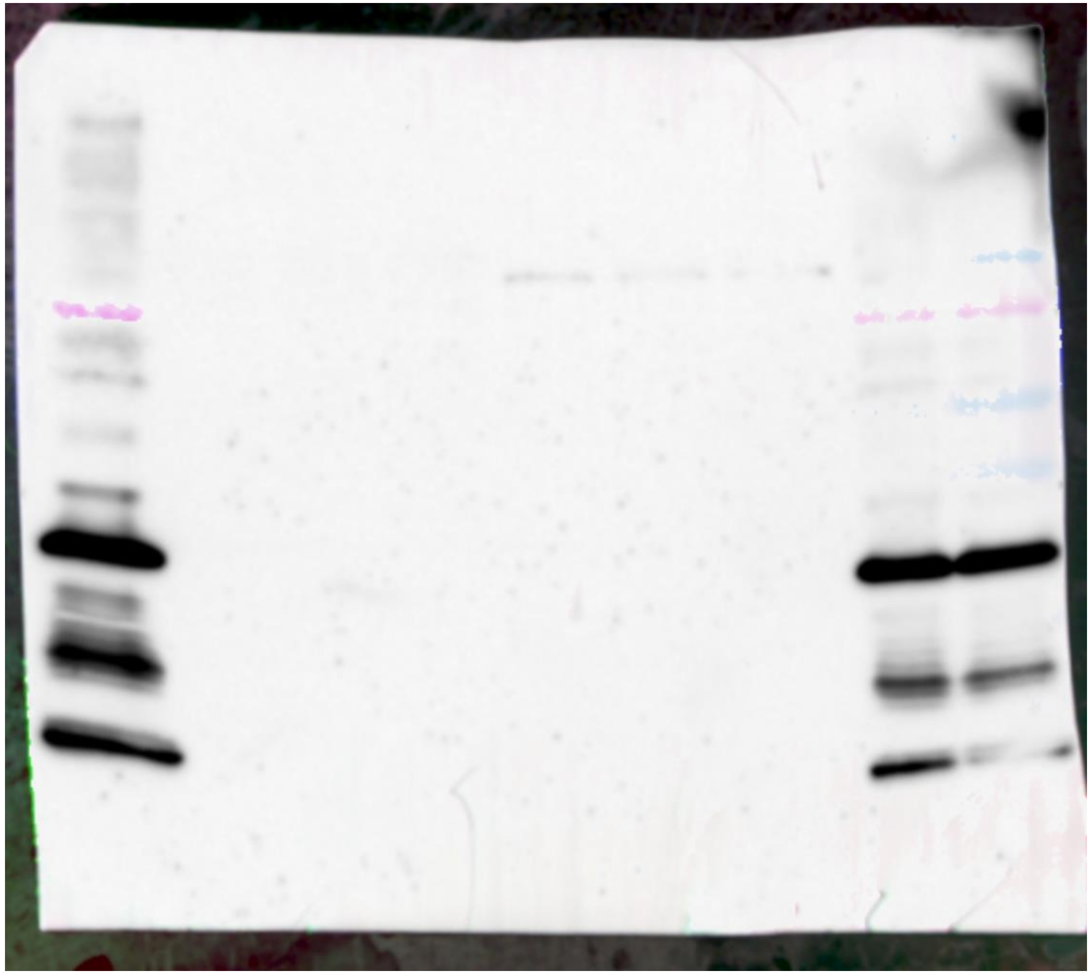

- Na^+/K^+-ATPase

Full unedited blot for Supplemental Figure 4B

GAPDH -

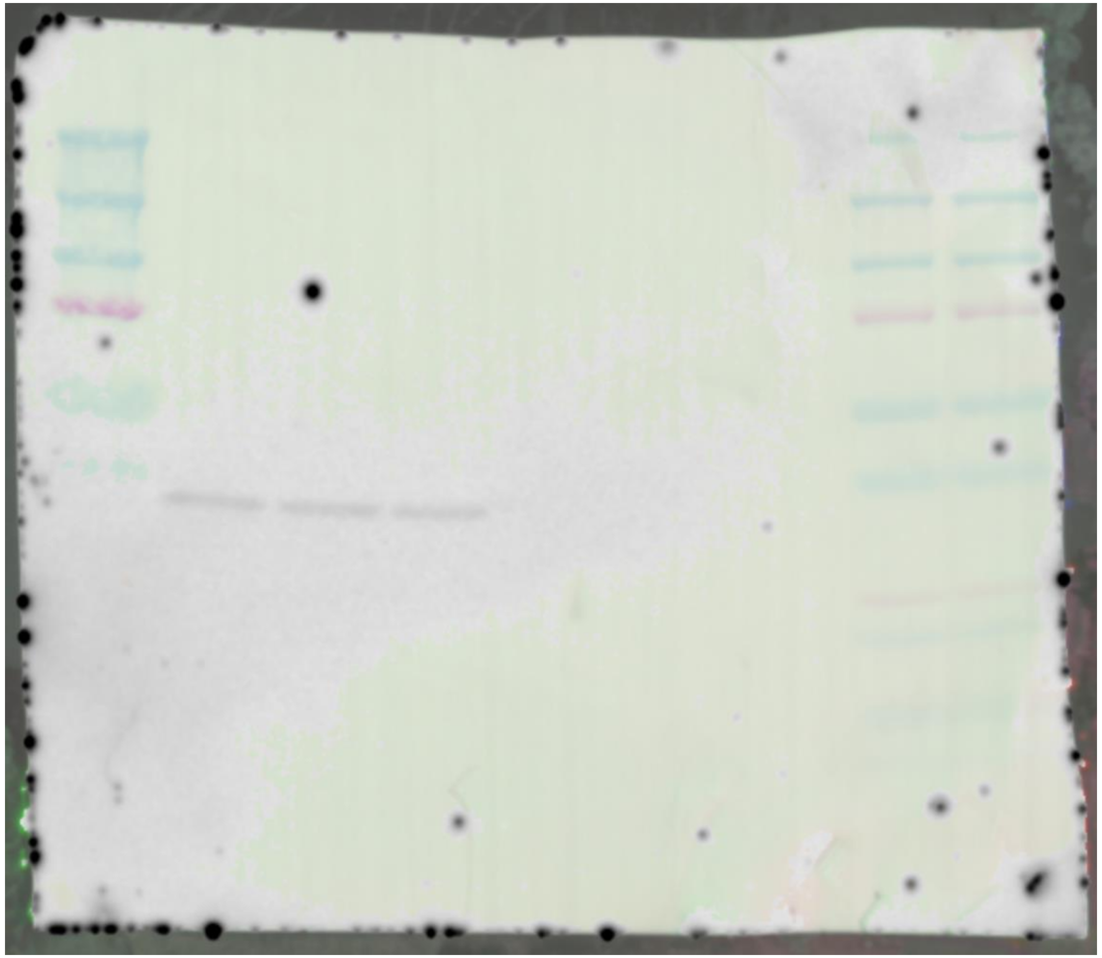

Supplement: Unedited blot and gel images [file jci-135-192011-s217.pdf]
